# Older adults’ sexual well-being and person-centered psychotherapy: A qualitative study

**DOI:** 10.1192/j.eurpsy.2021.1968

**Published:** 2021-08-13

**Authors:** S. Von Humboldt, J. Ribeiro-Gonçalves, I. Leal

**Affiliations:** William James Center For Research, ISPA-Instituto Universitário, Lisbon, Portugal

**Keywords:** Challenges, sexual well-being, older adults, person-centered psychotherapy

## Abstract

**Introduction:**

A qualitative study about older adults’ sexual well-being and person-centered psychotherapy

**Objectives:**

The objective of this study was to evaluate the main concerns related to sexual well-being revealed by older adults in person-centered therapy, using qualitative research.

**Methods:**

Interviews with 58 older participants, aged 65 to 82 years, living in the community were submitted to content analysis.

**Results:**

Eight main themes emerged from the results of the content analysis: Partner unavailability, family issues, physical changes due to aging, worries about hygiene, sexual dysfunctions, fear of physical abuse, communication issues and concerns about sexual transmitted diseases.

**Conclusions:**

This study was relevant towards identifying the challenges older adults feel regarding their sexual well-being, as shared in therapy. Older adults referred their greatest challenges to be partner unavailability, family issues and physical changes due to aging.

**Disclosure:**

No significant relationships.

